# Clinical factors associated with biased estimation of glomerular filtration rate: a cross-sectional study

**DOI:** 10.1093/ckj/sfag157

**Published:** 2026-05-22

**Authors:** Alexandre Lahens, Emmanuelle Vidal-Petiot, Nahid Tabibzadeh, Anne Boutten, François Rouzet, Timothée Fearon, François Vrtovsnik, Martin Flamant, Jimmy Mullaert

**Affiliations:** Physiology Department, Assistance Publique Hôpitaux de Paris, Hôpital Bichat-Claude Bernard, Paris, France; Institut Curie, INSERM, U1331 Computational Oncology, Saint-Cloud, France; Université Paris-Saclay, UVSQ, Montigny-le-Bretonneux, France; Physiology Department, Assistance Publique Hôpitaux de Paris, Hôpital Bichat-Claude Bernard, Paris, France; Université Paris Cité, INSERM U1148, LVTS, Paris, France; Physiology Department, Assistance Publique Hôpitaux de Paris, Hôpital Bichat-Claude Bernard, Paris, France; Université Paris Cité, Mecidine UFR, Paris, France; Unité Mixte de Recherche (UMR) S1138, Cordeliers Research Center, Paris, France; Biochemistry Department, Assistance Publique Hôpitaux de Paris, Hôpital Bichat-Claude Bernard, Paris, France; Université Paris Cité, Mecidine UFR, Paris, France; Department of Nuclear Medicine, Assistance Publique Hôpitaux de Paris, Hôpital Bichat-Claude Bernard, Paris, France; Physiology Department, Assistance Publique Hôpitaux de Paris, Hôpital Bichat-Claude Bernard, Paris, France; Université Paris Cité, Mecidine UFR, Paris, France; Department of Nephrology, Assistance Publique Hôpitaux de Paris, Hôpital Bichat-Claude Bernard, Paris, France; Center for Research on Inflammation, Université de Paris, INSERM U1149, Paris, France; Physiology Department, Assistance Publique Hôpitaux de Paris, Hôpital Bichat-Claude Bernard, Paris, France; Université Paris Cité, INSERM U1148, LVTS, Paris, France; Institut Curie, INSERM, U1331 Computational Oncology, Saint-Cloud, France; Université Paris-Saclay, UVSQ, Montigny-le-Bretonneux, France

**Keywords:** CKD-EPI equation, creatinine, creatinine clearance, cystatin C, GFR

## Abstract

**Background and hypothesis:**

Equations estimating glomerular filtration rate (GFR) based on creatinine and/or cystatin C incorporate demographic variables such as age and sex. However, clinical determinants may lead to substantial bias in GFR estimation. We aimed to identify clinical factors associated with biased GFR estimation with the Modification of Diet in Renal Disease, Chronic Kidney Disease-Epidemiology Collaboration, and European Kidney Function Consortium equations.

**Methods:**

In this retrospective cross-sectional study, we included patients referred for GFR measurement from March 2008 to February 2024 in the Physiology unit of Bichat Hospital, Paris, France. GFR was measured as the urinary clearance of a radio-isotopic tracer and the error of estimated GFR (eGFR), was expressed as log(eGFR/measured GFR) and analyzed with linear regression models.

**Results:**

Among 3838 patients (mean age 51 ± 15 years, mean measured GFR 59 ± 26 ml/min/1.73 m²), several clinical variables were associated with significant estimation error in the multivariable analysis. For all creatinine-based equations, underestimation occurred with HIV infection, high BMI, loop diuretics, and cotrimoxazole use, while overestimation occurred with younger age, female sex, lower BMI, history of kidney transplantation, and cirrhosis. For all cystatin C-based equations, underestimation was associated with older age, HIV infection, corticosteroid use, and history of kidney transplantation; overestimation was associated with younger age, female sex, and sub-Saharan African origin. Equations using both biomarkers performed better in conditions affecting each biomarker in opposite directions. By cumulating several conditions, bias can vary from −50% to more than +50% leading to a completely erroneous GFR estimation.

**Conclusion:**

Common clinical features are independently associated with biased GFR estimation that can be of high clinical relevance, especially when cumulated. Our results guide GFR evaluation by giving a qualitative and most importantly quantitative estimation of the expected bias depending on individual patient profile and comorbidities.

## Graphical Abstract

**Graphical Abstract:**
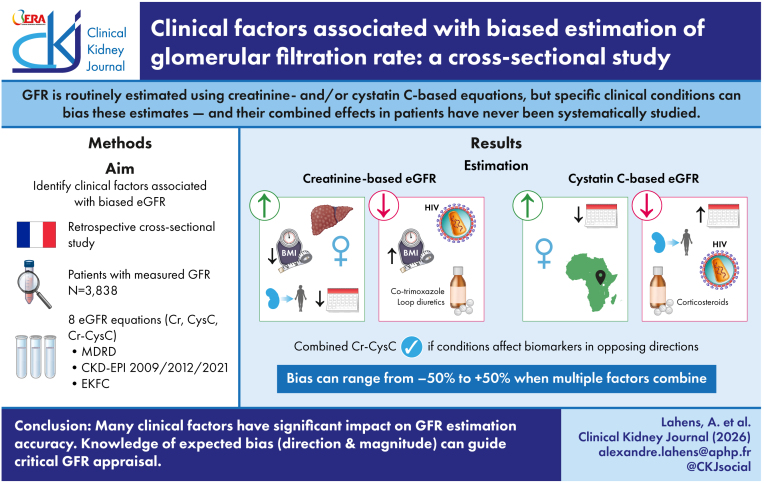

KEY LEARNING POINTS **What was known:**

- Glomerular filtration rate estimation relies on creatinine and/or cystatin C serum concentrations. In some conditions, their serum concentration may be altered by factors influencing non glomerular determinants of these biomarkers leading to false estimation. However, those conditions are always studied separately without consideration for comorbidities.

**This study adds:**

- We analyzed a large cohort of patients, of all chronic kidney disease spectrum with most common comorbidities and medications taken in this population and provided the specific impact of these comorbidities or medications on estimation bias in a multivariate analysis.

**Potential impact:**

- Clinicians that need to properly assess kidney function of complex patients will have a better appreciation of the estimations given by the equation, knowing their specific bias. It should help clinicians choose what equation is more accurate, or when ask for a GFR measurement.

## INTRODUCTION

Glomerular filtration rate (GFR) is the best available indicator of kidney function and is routinely estimated using equations based on endogenous filtration markers. Since Cockcroft & Gault formula in 1976 [1] several equations have been developed to estimate GFR, including the Modification of Diet in Renal Disease (MDRD) equation in 1999 [2], the Chronic Kidney Disease Epidemiology Collaboration (CKD-EPI) equations in 2009, 2012, and 2021 [3–5] and more recently the European Kidney Function Consortium (EKFC) equations in 2021 and 2023 [6, 7]. These equations incorporate plasma biomarkers, most commonly creatinine and more recently cystatin C, and demographic variables. Although these equations showed good performances in their developing population, their accuracy decreases in specific subgroups, due to determinants of biomarkers concentration that are independent of renal glomerular filtration. Creatinine production is driven by muscle mass, which varies widely among individuals, so that for the same measured GFR, individuals with different muscle mass will have different serum creatinine levels. This explains the inclusion of demographic variables such as age, sex, and sometimes ethnicity as proxies for muscle mass in eGFR equations. However, regression models incorporating measured GFR and demographic variables typically explain only approximately two-thirds of the variance in serum biomarker levels [6], underscoring the contribution of unmeasured or poorly characterized factors. For instance, it has been shown that GFR tends to be overestimated in patients with cirrhosis, largely due to a lower muscle mass than individuals of same age, sex, and ethnicity [8–11, 12]. In addition, tubular secretion and, to a lesser extent, intestinal excretion also affect serum creatinine and vary widely between individuals, influenced by drugs or diseases. As estimation equations account for average tubular secretion of creatinine, medications inhibiting tubular secretion such as trimethoprim-containing antibiotics lead to GFR underestimation [13, 14]. Inaccuracy in GFR estimation has also been reported in individuals with obesity [15] and in kidney transplant recipients [16, 17] likely due to multifactorial factors. Conditions influencing cystatin-C levels outside of renal function include age, sex, or diabetes [18] but are not as well described as for creatinine.

Most studies assessing the impact of specific disease states on the performance of estimating equations have been conducted in targeted subpopulation studies, often overlooking comorbidities and pharmacologic exposures that may exert cumulative or opposing effects on biomarker levels.

In this large, single-center study involving patients with various profiles of comorbidities and renal function who underwent gold-standard GFR measurement (using urinary clearance of a radio-isotopic tracer) and isotope dilution mass spectrometry (IDMS) traceable enzymatic creatinine and standardized cystatin C dosages, we aimed to identify clinical variables associated with systematic bias in routinely used GFR estimating equations. We performed both univariable and multivariable analyses to quantify these biases and improve understanding of their individual-level determinants beyond standard demographic adjustments.

## MATERIALS AND METHODS

### Patients

All consecutive patients with available and valid urinary GFR measurement and IDMS traceable enzymatic creatinine dosage in the physiological laboratory of Bichat Hospital (AP-HP) from March 2008 and February 2024 were included in this study. Patients were informed of the study and gave written consent for scientific use of anonymized data. This study was approved by the local ethics committee (Institutional Review Board–IRB 00006477). GFR was measured for several reasons, including kidney donor candidates, follow-up after kidney transplantation, potential inaccurate estimation, or metabolic evaluation in different clinical contexts (chronic kidney disease, kidney stones, ionic disorder, HIV infection, potential drug-induced toxicity…). In case of repeated GFR measurement during the study period, only the first complete available evaluation was kept for this study.

### Data collection

Age, sex, ethnicity (self-declared), weight, height, medication, and comorbidities were prospectively collected. Medication collected were steroids, cotrimoxazole (usually low-dose prophylaxis), anti-diabetics (oral and insulin) and anti-hypertensive drugs. In the vast majority of patients, steroids were given for stable chronic disease or kidney transplantation, hence doses of 10 mg or less.

### GFR measurement

GFR was measured using the urinary and plasma clearance of a radio-isotope, in all patients. The radio-isotope was 51Cr-labeled ethylenediaminetetraacetic acid (51Cr-EDTA) from 2008 to February 2020 and Technetium-99m-diethylenetriaminepentaacetic acid (99mTc-DTPA) from 2020 to 2024. Each syringe was weighed before and after injection, in order to calculate the injected amount of the tracer. After a 90-min resting period to allow equilibrium of the tracer in its distribution volume, urine was collected every 30 min for six consecutive periods, hence for a total of 270 min after injection. Blood samples were collected from the arm contralateral to the injection at the mid-time of each period. Activity of urinary and plasma samples together with standards were measured with the Wallac Wizard 3″1480 (PerkinElmer) following gamma counter. Urinary clearance was calculated as the average of the six mini-equilibrium clearances, and used as gold-standard mGFR value, so as to avoid any inaccuracy resulting from plasma clearance, such as in patients with cirrhosis or extreme BMI [19, 20]. In case of irregular voiding, the visit was excluded from all analyses (Fig. S1). Each measurement was validated by the physician.

Urinary clearance of creatinine was measured simultaneously to mGFR, using the same urine samples.

Body surface area was calculated from weight and height using the Du Bois and Du Bois formula [21] and all GFR values were normalized to 1.73 m².

### GFR estimations

Estimated GFR was calculated from four creatinine-based equations [the MDRD as simplified in 2006 (2), the CKD-EPI equations published in 2009 and 2021 (3,5), and the EKFC equations (6)], two cystatin C-based equations [the CKD-EPI equation published in 2012 (4) and the 2023 EKFC (7)], and two combined (creatinine and cystatin C) equations [CKD-EPI and EKFC, respectively published in 2021 and 2023 (5,7)]. Formulas of these equations are detailed in Table S1.

For EKFC_cr_ equation, a Q factor depending on sex and ethnic group was used, and for EKFC_cys_ a Q factor depending on sex.

Plasma creatinine was measured with enzymatic assays traceable to the IDMS reference method, ECREA® on a Dimension Vista® analyzer (Siemens Healthineers®) until 13/09/2022, then CREP2® on a Cobas 503® analyzer (Roche Diagnostic®). Plasma cystatin C was measured with standardized assays according to the International Federation for Clinical Chemistry and Laboratory Medicine (IFCC reference material ERM-DA471/IFCC) [22]. The nephelometric CYSC® immunoassay, on a Dimension Vista® analyzer (Siemens Healthineers®) was used until September 2022, replaced by the turbidimetric assay CYSC2®, on a Cobas 503® analyzer (Roche Diagnostic®) thereafter.

### Statistical methods

Continuous variables were reported as mean and standard deviation (SD) if they were normally distributed and as median and interquartile range (IQR, 25th to 75th percentile) otherwise, and categorical variables were reported as number and percentage.

For every equation, we calculated the absolute bias (eGFR-measured GFR) and the relative bias (eGFR-measured GFR/measured GFR) and reported their median and (IQR). We then calculated the relative bias for each modality of clinical variables and compared the bias between modalities with a univariable linear regression. In order to accommodate multiple testing issues, we considered a *P*-value below .001 and .05 as statistically significant for the univariable and multivariable analysis respectively.

For this analysis, we used a univariable linear regression model, where the dependent variable and the explanatory variables were the error of estimation, defined as log (eGFR/measured GFR), and the clinical factors, respectively.

For multivariable linear regression, the variable selection was based on a priori selection of potential confounders, and were finally: age (categorized in: <40, 40–60, and >60 years), sex, ethnicity, BMI (categorized in: <18.5, 18.5–30, and >30 kg/m²), cirrhosis, history of hypertension, diabetes, HIV, kidney transplant recipients, use of corticosteroids, cotrimoxazole, and loop diuretics. Age and BMI were analyzed as categorical variables due to their non-linear relationship with eGFR bias.

Measured GFR was not included in the main multivariable analysis as we intended to give results useable for clinicians that would not have the information on measured GFR. A sensitivity analysis was then made including measured GFR.

For both uni and multivariable analysis, we reported the exponential of the regression coefficient minus one in %, representing the relative effect on eGFR/measured GFR associated with the variable, and its 99.9 or 95% confidence intervals (CI).

## RESULTS

Between 18 March 2008 and 15 February 2024, 3838 patients were admitted to the physiology unit of Bichat Hospital for a first GFR measurement with simultaneous IDMS traceable creatinine dosage, among them 2637 (69%) also had a cystatin C dosage.

Baseline clinical characteristics of the population are reported in Table1. Mean age was 51 years, 24% reported a sub-Saharan African origin, 36% were kidney transplant recipients, 67% had hypertension, and 19% had diabetes. Regarding kidney function, mean measured GFR was 59 ml/min/1.73 m², 14% of patients had normal GFR (≥90 ml/min/1.73 m²), 32% had mildly decreased GFR (60–89 ml/min/1.73 m²), and 54% had chronic kidney disease of stage 3 or more (<60 ml/min/1.73 m²).

**Table 1:** Patient characteristics.

Characteristic	Total population	Creatinine and cystatin C	Creatinine only
Patients, *n*	3838	2637	1201
Female, *n (%)*	1574 (41)	1098 (42)	476 (40)
Age, *n* (%)			
<40 years	994 (26)	702 (27)	292 (24)
40–60 years	1797 (47)	1220 (46)	577 (48)
>60 years	1047 (27)	715 (27)	332 (28)
Ethnic group [African origin (self-reported)], *n (%)*	910 (24)	663 (25)	247 (21)
Clinical characteristics			
Mean body mass index (SD), kg/m²	26.0 (5.3)	25.9 (5.3)	26.2 (5.2)
Mean systolic blood pressure (SD), mmHg	130.7 (18.6)	129.3 (18.8)	133.9 (17.8)
Mean diastolic blood pressure (SD), mmHg	75.7 (11.6)	75.4 (11.0)	76.5 (12.8)
Comorbidities, *n (%)*			
Diabetes	746 (19)	537 (20)	209 (17)
Hypertension	2578 (67)	1652 (63)	926 (77)
Other solid organ transplant recipient	1587 (41)	1042 (40)	545 (45)
Kidney transplant recipient	1388 (36)	927 (35)	461 (38)
Kidney donor candidate	410 (11)	291 (11)	119 (9.9)
Solitary kidney	167 (4.4)	73 (2.8)	94 (7.8)
Cirrhosis	182 (4.7)	107 (4.1)	75 (6.2)
HIV infection	340 (8.9)	274 (10)	66 (5.5)
Medication use, *n (%)*			
Antihypertensive drug	2375 (62)	1520 (58)	855 (71)
ACE inhibitor	574 (15)	297 (11)	277 (23)
ARB	796 (21)	474 (18)	322 (27)
Beta blocker	1277 (33)	819 (31)	458 (38)
Calcium channel inhibitor	1297 (34)	888 (34)	409 (34)
Loop Diuretic	446 (12)	252 (9.6)	194 (16)
Thiazide or thiazide-like diuretic	287 (7.5)	167 (6.3)	120 (10)
K-sparing diuretic	77 (2)	59 (2.2)	18 (1.5)
iSGLT-2	19 (0.5)	19 (0.72)	0 (0)
Calcineurin inhibitor	1487 (39)	973 (37)	514 (43)
Trimethoprim-sulfamethoxazole	3008 (78)	1986 (75)	1022 (85)
Corticosteroid	1113 (29)	823 (31)	290 (24)
Laboratory			
Mean plasma creatinine level (SD), µmol/l	125.0 (64.5)	119.3 (59.6)	137.4 (72.6)
Mean plasma Cystatin C level (SD), mg/l	1.4 (0.6)	1.4 (0.6)	
Kidney function, mL/min per 1.73 m²			
Mean urinary creatinine clearance (SD)	75.1 (32.0)	78.3 (32.1)	68.0 (30.6)
Mean measured GFR (SD)	59.3 (26.0)	62.2 (25.7)	53.0 (25.6)
CKD stages, *n (%)*			
Stage I	532 (14)	413 (16)	119 (9.9)
Stage II	1227 (32)	945 (36)	282 (23)
Stage III	1564 (41)	988 (37)	576 (48)
Stage IV/V	515 (13)	291 (11)	224 (19)

SD, standard deviation; HIV, human immunodeficiency virus; ACE, angiotensin converting enzyme; ARB, angiotensin receptor blockers; iSGLT-2, inhibitor of sodium-glucose cotransporter type 2; GFR, glomerular filtration rate; CKD, chronic kidney disease.

### Overall performance of GFR estimating equations

Absolute and relative biases of GFR estimation are reported in Table 2. The MDRD_cr_ equation had the lowest bias among creatinine-based formulas (+1.8 ml/min/1.73 m² and +3.6% absolute and relative bias, respectively), followed by the EKFC_cr_ equation (+3.5 ml/min/1.73 m² and +6.7% absolute and relative bias, respectively), while the largest bias was observed with the CKD-EPI_cr_ equations (2009 and 2021). The CKD-EPI_cr_ 2021 equation overestimated GFR to a greater extent than the 2009 equation, respectively +12.1 and +10.4% of relative bias.

**Table 2:** Estimated glomerular filtration rate, and bias and relative bias compared with measured glomerular filtration rate, for every equation tested.

	GFR estimation	Error compared with measured GFR	
	Median value ml/min per 1.73 m² (IQR)	Median bias mL/min/1.73 m² (IQR)	Median relative bias % (IQR)
Creatinine-based equations (*N* = 3838)			
MDRD_cr_	58 (41; 80)	+2 (−5; 10)	+4 (−9; 20)
CKD-EPI 2009_cr_	63 (43; 88)	+5 (−2; 14)	+10 (−4; 27)
CKD-EPI 2021_cr_	64 (44; 90)	+6 (−1; 15)	+12 (−2; 29)
EKFC_cr_	61 (42; 84)	+4 (−3; 11)	+7 (−6; 22)
Cystatin C-based equation (*N* = 2637)			
CKD EPI_cys_	59 (39; 87)	–0 (−8; 9)	–1 (−15; 16)
EKFC_cys_	62 (43; 87)	+3 (−5; 10)	+5 (−8; 19)
Creatinine and cystatin C-based equation (*N* = 2637)			
CKD EPI 2021_cr-cys_	64 (44; 89)	+3 (−3; 11)	+6 (−6; 20)
EKFC_cr-cys_	63 (46; 86)	+3 (−3; 9)	+6 (−5; 18)

GFR, glomerular filtration rate; MDRD, modification of diet in renal disease; CKD-EPI, chronic kidney disease epidemiology collaboration; EKFC, European kidney function consortium. Bias is defined as the difference between mGFR and eGFR; relative bias is defined as mGFR-eGFR/mGFR.

IQR, interquartile range (refers to 25th to 75th percentile).

Cystatin C-based equations showed overall minimal bias. CKD-EPI_cys_ had a near-zero bias of –0.5% of relative bias. Finally, regarding combined creatinine and cystatin C-based equations, the lowest bias was observed for the EKFC_cr-cys_ equation (+3 ml/min/1.73 m² and +5.7% relative bias).

### Bias depending on clinical variables

**Figure 1:**
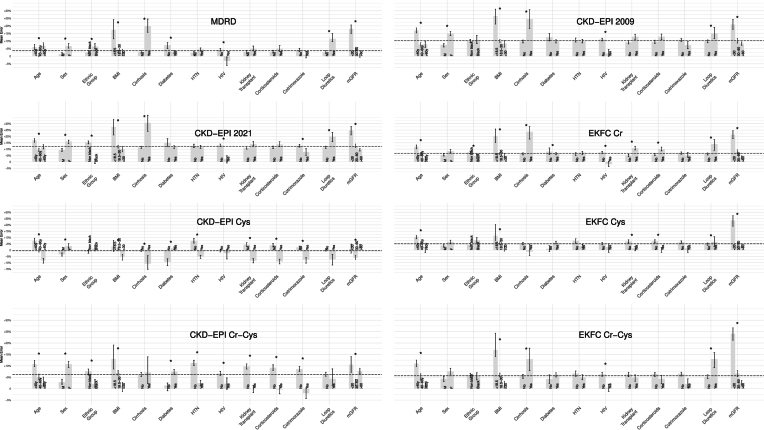
Relative bias defined as (eGFR-mGFR)/mGFR, according to clinical categories. BMI, body mass index; HTN, hypertension; HIV, human immunodeficiency virus; mGFR, measured GFR.

We first report on Fig. 1 relative bias according to different modalities of each variable tested. For creatinine-based equations, we observed a great overestimation for cirrhosis, low BMI, loop diuretics, and low measured GFR (<30 ml/min/1.73 m²); and an underestimation for HIV infection and cotrimoxazole use. For cystatin C-based equation, there was an underestimation with multiple variables for CKD-EPI_cys_ equation: HIV, diabetes, kidney transplantation, corticosteroids, and cotrimoxazole use, whereas for EKFC_cys_ equation we mainly observed an overestimation for low measured GFR. For combined equations, we also observed that CKD-EPI_cr-cys_ equations were more sensitive to clinical variables, but less to measured GFR, with an overestimation associated with the absence of certain comorbidities as diabetes, hypertension, kidney transplantation, corticosteroids, and cotrimoxazole use, probably explained by the overall overestimation of CKD-EPI_cr-cys_.

Biases stratified by clinical variables in univariable and multivariable analyses are shown in Table 3.

**Table 3:** Relative effect on eGFR/mGFR of each clinical parameter in uni and multivariable analyses, per equation.

Panel A								
	MDRD_cr_		CKD EPI 2009_cr_		CKD EPI 2021_cr_		EKFC_cr_	
	Unadjusted effect, *% [99.9% CI]*	Adjusted effect, % [95% CI]	Unadjusted effect, *% [99.9% CI]*	Adjusted effect, % [95% CI]	Unadjusted effect, *% [99% CI]*	Adjusted effect, % [95% CI]	Unadjusted effect, *% [99.9% CI]*	Adjusted effect, % [95% CI]
Age 40–59 y	Ref	Ref	Ref	Ref	Ref	Ref	Ref	Ref
Age <40 y	4.5 [2.6; 6.3]	5.4 [3.5; 7.3]	8.1 [6.3; 10.0]	8.8 [6.9; 10.8]	6.7 [4.8; 8.6]	7.1 [5.2; 9.0]	4.4 [2.6; 6.2]	5.4 [3.6; 7.2]
Age ≥60 y	5.7 [3.9; 7.6]	4.3 [2.4; 6.2]	−0.2 [−1.9; 1.5]	−1.5 [−3.2; 0.3]	2.7 [0.9; 4.5]	−0.0 [−1.8; 1.8]	−3.9 [−5.5; −2.3]	−6.0 [−7.6; −4.3]
Sex [male]	Ref	Ref	Ref	Ref	Ref	Ref	Ref	Ref
Sex [female]	4.0 [2.5; 5.6]	4.3 [2.7; 5.8]	5.9 [4.4; 7.5]	5.9 [4.4; 7.5]	5.5 [4.0; 7.1]	5.4 [3.9; 6.9]	2.2 [0.8; 3.7]	2.4 [1.0; 3.9]
Non-sub-Saharan African origin	Ref	Ref	Ref	Ref	Ref	Ref	Ref	Ref
Sub-Saharan African origin	3.4 [1.7; 5.2]	6.2 [4.3; 8.1]	0.0 [−1.6; 1.8]	2.1 [0.3; 3.8]	−13.6 [−15.0; −12.1]	−11.8 [−13.3; −10.3]	−3.3 [−4.9; −1.7]	−2.2 [−3.8; −0.5]
BMI 18.5–29.9 kg/m²	Ref	Ref	Ref	Ref	Ref	Ref	Ref	Ref
BMI <18.5 kg/m²	15.1 [11.0; 19.3]	15.2 [11.2; 19.4]	15.8 [11.8; 20.0]	13.6 [9.7; 17.6]	15.2 [11.1; 19.5]	13.4 [9.5; 17.4]	14.2 [10.3; 18.1]	12.9 [9.2; 16.8]
BMI > 30 kg/m²	−0.7 [−2.5; 1.2]	−3.0 [−4.8; −1.1]	−1.8 [−3.6; −0.0]	−2.8 [−4.6; −1.0]	−2.0 [−3.8; −0.1]	−2.7 [−4.5; −0.9]	−1.8 [−3.5; −0.0]	−2.7 [−4.4; −0.9]
Absence of comorbidity/medication	Ref	Ref	Ref	Ref	Ref	Ref	Ref	Ref
Cirrhosis	19.2 [15.2; 23.3]	20.1 [16.1; 24.2]	15.3 [11.5; 19.3]	19.0 [15.2; 23.0]	18.8 [14.8; 22.9]	18.9 [15.0; 22.8]	16.5 [12.7; 20.3]	19.1 [15.3; 22.9]
HIV infection	−8.1 [−10.5; −5.8]	−6.2 [−8.6; −3.8]	−8.3 [−10.6; −6.0]	−5.3 [−7.6; −2.8]	−11.2 [−13.5; −8.9]	−5.1 [−7.5; −2.7]	−8.6 [−10.8; −6.4]	−5.4 [−7.6; −3.0]
Hypertension	2.3 [0.7; 3.9]	0.5 [−1.3; 2.3]	−0.4 [−1.9; 1.1]	0.3 [−1.5; 2.1]	−0.3 [−1.9; 1.3]	0.8 [−0.9; 2.6]	1.5 [0.0; 3.1]	1.6 [−0.2; 3.3]
Kidney transplant recipient	1.2 [−0.3; 2.7]	3.2 [0.9; 5.6]	2.4 [0.8; 3.9]	4.9 [2.5; 7.2]	1.2 [−0.3; 2.8]	5.0 [2.7; 7.4]	4.3 [2.8; 5.8]	6.1 [3.8; 8.4]
Diabetes	4.5 [2.6; 6.5]	2.9 [0.8; 4.9]	2.1 [0.3; 4.0]	2.7 [0.7; 4.7]	2.5 [0.6; 4.4]	2.8 [0.9; 4.8]	2.8 [1.0; 4.6]	2.9 [1.0; 4.8]
Corticosteroid use	1.7 [0.1; 3.3]	1.9 [−0.4; 4.3]	2.3 [0.7; 3.9]	1.9 [−0.4; 4.2]	0.8 [−0.9; 2.4]	1.9 [−0.3; 4.2]	3.5 [1.9; 5.1]	2.2 [−0.0; 4.4]
Cotrimoxazole use	−3.7 [−5.6; −1.6]	−7.1 [−9.3; −4.9]	−3.3 [−5.2; −1.3]	−7.4 [−9.6; −5.2]	−4.3 [−6.3; −2.3]	−7.4 [−9.6; −5.3]	−1.6 [−3.6; 0.3]	−7.3 [−9.3; −5.1]
Loop diuretic use	10.3 [7.8; 12.8]	9.6 [7.0; 12.2]	5.9 [3.5; 8.2]	7.8 [5.3; 10.4]	7.5 [5.1; 10.0]	8.3 [5.8; 10.8]	7.8 [5.5; 10.2]	10.1 [7.6; 12.6]
Panel B								
	CKD EPI_cys_				EKFC_cys_			
	Unadjusted effect, *% [99.9%]*		Adjusted effect, *% [95%]*		Unadjusted effect, *% [99.9%]*		Adjusted effect, *% [95%]*	
Age 40–59 y	Ref		Ref		Ref		Ref	
Age <40 y	7.8 [5.6; 10.1]		5.8 [3.6; 8.0]		7.0 [4.9; 9.0]		6.4 [4.3; 8.6]	
Age ≥60 y	−6.3 [−8.2; −4.4]		−5.4 [−7.4; −3.4]		−2.5 [−4.4; −0.7]		−3.6 [−5.5; −1.6]	
Sex [male]	Ref		Ref		Ref		Ref	
Sex [female]	6.0 [4.1; 7.9]		4.4 [2.6; 6.3]		2.4 [0.7; 4.0]		1.8 [0.1; 3.5]	
Non-sub-Saharan African origin								
Sub-Saharan African origin	3.0 [1.0; 5.1]		7.0 [4.9; 9.2]		3.0 [1.1; 4.9]		5.4 [3.4; 7.5]	
BMI 18.5–29.9 kg/m²	Ref		Ref		Ref		Ref	
BMI <18.5 kg/m²	6.7 [2.3; 11.3]		5.1 [1.0; 9.5]		8.9 [4.8; 13.1]		8.6 [4.5; 12.9]	
BMI >30 kg/m²	−5.6 [−7.7; −3.4]		−6.1 [−8.2; −4.0]		−3.0 [−5.0; −1.0]		−4.8 [−6.8; −2.7]	
Absence of comorbidity/medication	Ref		Ref		Ref		Ref	
Cirrhosis	−8.5 [−12.5; −4.4]		−7.6 [−11.4; −3.7]		−2.9 [−6.7; 1.1]		−3.2 [−7.0; 0.8]	
HIV infection	−2.1 [−4.9; 0.7]		−6.8 [−9.3; −4.1]		−3.6 [−6.1; −1.0]		−6.1 [−8.6; −3.5]	
Hypertension	−10.7 [−12.2; −9.1]		−1.7 [−3.7; 0.3]		−3.1 [−4.6; −1.5]		2.2 [0.2; 4.2]	
Kidney transplant recipient	−12.1 [−13.7; −10.6]		−7.5 [−10.4; −4.5]		−5.4 [−7.0; −3.9]		−3.7 [−6.6; −0.7]	
Diabetes	−8.9 [−10.8; −6.9]		−2.0 [−4.2; 0.4]		−3.2 [−5.1; −1.3]		−0.1 [−2.3; 2.2]	
Corticosteroid use	−12.9 [−14.5; −11.3]		−8.4 [−11.1; −5.6]		−6.8 [−8.4; −5.2]		−6.4 [−9.1; −3.6]	
Cotrimoxazole use	−9.7 [−11.9; −7.5]		0.4 [−2.5; 3.3]		−4.4 [−6.6; −2.3]		0.2 [−2.5; 3.0]	
Loop diuretic use	−5.7 [−8.5; −2.9]		2.6 [−0.5; 5.8]		4.8 [2.0; 7.7]		10.2 [7.1; 13.5]	
Panel C								
	CKD EPI_cr-cys_				EKFC_cr-cys_			
	Unadjusted effect, % [99.9%]		Adjusted effect, % [95%]		Unadjusted effect, % [99.9%]		Adjusted effect, % [95%]	
Age 40–59 y	Ref		Ref		Ref		Ref	
Age <40 y	5.6 [3.8; 7.4]		4.3 [2.5; 6.1]		5.6 [3.8; 7.4]		5.8 [4.0; 7.7]	
Age ≥60 y	−1.0 [−2.7; 0.7]		−1.3 [−3.0; 0.5]		−2.2 [−3.8; −0.6]		−4.0 [−5.7; −2.3]	
Sex [male]	Ref		Ref		Ref		Ref	
Sex [female]	6.2 [4.7; 7.7]		5.1 [3.6; 6.6]		2.2 [0.8; 3.6]		2.1 [0.6; 3.5]	
Non-sub-Saharan African origin	Ref		Ref		Ref		Ref	
Sub-Saharan African origin	−5.4 [−6.9; −3.8]		−1.9 [−3.5; −0.3]		−0.6 [−2.2; 1.0]		1.5 [−0.2; 3.2]	
BMI 18.5–29.9 kg/m²	Ref		Ref		Ref		Ref	
BMI <18.5 kg/m²	9.1 [5.4; 12.9]		7.9 [4.3; 11.6]		12.6 [9.0; 16.4]		11.9 [8.3; 15.7]	
BMI >30 kg/m²	−4.3 [−6.0; −2.5]		−5.2 [−6.9; −3.4]		−2.3 [−4.1; −0.6]		−4.1 [−5.8; −2.3]	
Absence of comorbidity/medication	Ref		Ref		Ref		Ref	
Cirrhosis	3.4 [−0.3; 7.2]		2.3 [−1.2; 5.9]		7.8 [4.1; 11.6]		7.8 [4.2; 11.6]	
HIV infection	−5.9 [−8.0; −3.7]		−6.2 [−8.4; −4.0]		−6.0 [−8.1; −3.9]		−5.7 [−7.9; −3.5]	
Hypertension	−7.4 [−8.7; −6.1]		−1.6 [−3.2; 0.1]		−0.8 [−2.2; 0.6]		1.7 [0.0; 3.4]	
Kidney transplant recipient	−8.8 [−10.1; −7.4]		−4.5 [−7.0; −2.0]		−1.2 [−2.6; 0.2]		−0.4 [−3.0; 2.2]	
Diabetes	−4.5 [−6.1; −2.8]		−0.0 [−1.9; 1.9]		0.3 [−1.4; 2.0]		1.7 [−0.2; 3.7]	
Corticosteroid use	−8.9 [−10.2; −7.5]		−3.9 [−6.3; −1.5]		−1.8 [−3.3; −0.3]		−1.5 [−4.0; 0.9]	
Cotrimoxazole use	−8.7 [−10.5; −6.9]		−2.7 [−5.0; −0.3]		−2.8 [−4.7; −0.9]		−3.1 [−5.4; −0.8]	
Loop diuretic use	0.4 [−2.0; 2.8]		5.3 [2.7; 8.0]		8.9 [6.4; 11.4]		13.1 [10.3; 16.0]	

Panel A: Creatinine-based equations, Panel B: Cystatin C-based equations, Panel C: Creatinine and cystatin C-based equations.

The unadjusted effect represents the exponential of the regression coefficient minus 1 associated with the variable of the linear regression of log[eGFR/mGFR] in an univariable analysis, and the adjusted effect represents the exponential of the regression coefficient minus 1 with the variable of the linear regression of log [eGFR/mGFR] in a multivariable analysis. In the multivariable model, covariates were to age, sex, ethnicity, BMI, cirrhosis, hypertension, treated diabetes, HIV, kidney transplant recipients, use of corticosteroids, cotrimoxazole, and loop diuretics.

Values reported in the table correspond to the multiplicative factor that should be applied to the ratio eGFR/mGFR of the reference category to obtain the same ratio in the category of interest (with their 99.9 or 95% confidence intervals). For example, the value 5.6% for age <40 of the CKD-EPI_cr-cys_ equation (panel C) means that the ratio eGFR/mGFR is, on average, multiplied by 1.056 if a patient of age <40 is considered instead of a patient of age 40–59 y.

*Grey cases highlight that confidence interval does not include 0

#### Creatinine-based equations

Age <40 years was independently associated with GFR overestimation both in uni- and multivariable analyses across every equation. Conversely, age >60 years yielded equation-specific effects with an overestimation with MDRD_cr_ but an underestimation with EKFC_cr_ that showed an underestimation of –6%.

Female sex was consistently associated with slight GFR overestimation for every equation, even EKFC_cr_ (with sex-specific Q-factor). Sub-Saharan African origin was associated with a +6% overestimation for MDRD_cr_ (which uses a correction factor of 1.21), was nearly unbiased in the CKD-EPI 2009 equation (which uses a correction factor of 1.16), and was associated with a large underestimation in the CKD-EPI 2021 race-free equation (–12% compared to GFR estimation in patients of non-sub-Saharan African origin). Low BMI was a significant predictor of marked (+10 to +15%) overestimation in adjusted analyses while high BMI was associated with an underestimation of approximatively –5%. Cirrhosis was associated with a marked (+15% to +20%) GFR overestimation. Use of loop diuretics was also associated with a clinically significant overestimation of nearly +10% with every equation, history of kidney transplantation and diabetes were also associated with a slight overestimation between +3 and +6% in adjusted analyses. HIV infection and cotrimoxazole use were associated with GFR underestimation (–5 to –9%).

#### Cystatin C based equations

Age <40 years, sub-Saharan African origin and low BMI, were associated with modest GFR overestimation, as observed for creatinine-based equations. HIV, corticosteroid use, and kidney transplantation were independently associated with GFR underestimation of approximately –5 to –10%. Cirrhosis was associated with a marked and statistically significant underestimation for CKD-EPI_cys_ [–7.6 95% CI ([–11.4; –3.7)] whereas the underestimation of EKFC_cys_ did not reach significance (–3.2 [–7.0; 0.8]). Use of loop diuretics was associated with an overestimation of +10% with EKFC_cys_ only.

#### Combined equations

Combined CKD-EPI and EKFC equations still exhibited biases, but often less pronounced with opposing creatinine and cystatin C-related biases often attenuating one another. For instance, the bias was not clinically relevant for patients having cirrhosis with CKD-EPI_cr-cys_ combined equations. However significant bias remained in some subgroups (e.g. age <40 years, female sex, BMI <18.5 kg/m², and >30 kg/m², cotrimoxazole use and history of HIV infection).

#### Sensitive analysis

In Table S2, are presented the results of the multivariable analysis including measured GFR as a covariate. First, we observed a strong GFR effect on low measured GFR with a marked overestimation under 30 ml/min/1.73 m² (between +10 and +22%) with every equation. Then, it confirmed the impact of clinical variables, independently of measured GFR, except for low BMI and cystatin-C based equations, suggesting that the overestimation observed in Table 3 is in fact due to lower GFR is this sub-population.

## DISCUSSION

In this large, single-center cohort of 3838 patients with gold-standard GFR measurement and enzymatic creatinine dosage, including 2637 who also had cystatin C dosage, we assessed GFR estimation bias associated with clinical variables across commonly used equations. Our findings demonstrate that GFR estimation is significantly impacted by several clinical and demographic factors, including by parameters accounted for in the equations (i.e. age, sex, and ethnicity). Our results show the magnitude and direction of bias for all these factors, across creatinine-based, cystatin C–based, and combined equations.

### Variables included in the equations

One of the main findings of our study is that variables included in GFR equations—such as age, sex, and ethnicity—are still independently associated with estimation bias. This suggests that either the models are not perfectly fitted, or that the coefficients associated with the variable may be valid in the development cohorts and prone to error in validation populations.

For example, all equations overestimated GFR in patients under 40, and several of them underestimated it in older patients, suggesting that neither linear age adjustments (CKD-EPI) nor piecewise models (EKFC) fully capture biomarker level evolution with age. It is interesting to note that both models have their limitations: EKFC_cr_, which models a stable GFR for a given creatinine value before 40 years old, leads to a more accurate estimation that CKD-EPI_cr_ in younger patients, but it underestimates GFR in older patients suggesting that its age coefficient (0.990 vs 0.993 for CKD-EPI), applied after 40 years, may be slightly too strong.

An interesting finding of our study is the higher bias observed in women, especially with the CKD-EPI equations. This may reflect variations in the differential effect of sex on muscle masses in the different populations. However, it is interesting to note that the CKD-EPI (creatinine-derived) equation reported an overestimation of measured GFR in its own validation cohort, a finding further confirmed in several external studies [23, 24]. Whether this overestimation differed according to sex was not evaluated. Our study confirms the overall overestimation (particularly with CKD-EPI_cr_), which seems to be, at least partly, sex-dependent.

Concerning cystatin C–based equations although they are considered sex-independent, our results show that sex may be an extra-renal determinant of this biomarker. In line with this, in our study, we applied the sex-specific Q factors indicated in the original EKFC_cys_ publication (0.86 mg/l for men and 0.79 mg/l for women) which mathematically explains the markedly smaller sex-related bias than with the CKD-EPI_cys_ equation, which does not account for sex.

The race factor, though controversial and excluded from the most recent equations [5, 7], did reduce bias in patients of self-declared sub-Saharan African origin in our cohort. Removing the race coefficient (e.g. in CKD-EPI 2021) increased bias dramatically by up to +15%. Of note, even cystatin C–based equations showed residual bias between patients of sub-Saharan African origin and others.

### Variables not included in the equations

Beyond demographic variables, we identified several clinical factors independently associated with GFR estimation bias.

For creatinine-based equations, HIV infection and cotrimoxazole use were associated with underestimation—likely due to known inhibition of tubular creatinine secretion by cotrimoxazole and certain anti-HIV drugs (e.g. tenofovir or dolutegravir) or HIV-associated proximal tubular dysfunction. High BMI was associated with a slight underestimation, possibly reflecting higher creatinine production related to higher muscle mass with increasing BMI, but also possibly related to the limited performances of the equations (which generate indexed GFR values) in the extreme ranges of BMI. In contrast, cirrhosis, kidney transplantation, diabetes and low BMI, were all linked to overestimation. These conditions may reflect reduced muscle mass compared with patients of the same age and sex in whom equations were developed. Use of loop diuretics was also associated with a marked overestimation of nearly +10% with every creatinine-based equation, this is, to our knowledge, the first time it has been described, further studies might be of interest to determine whether it is due to a difference in muscle mass or in tubular secretion, as we shown in our sensitivity analysis that it was not due to lower GFR.

For cystatin C–based equations, HIV infection, corticosteroids use, and kidney transplantation were associated with underestimation. BMI markedly influenced bias, with a +5%–10% overestimation at low BMI and an underestimation of similar magnitude at high BMI. This indicates that body mass is correlated with cystatin C production, possibly in part due to the effects of adiposity and obesity-related inflammation on the biomarker. Moreover, as for creatinine-derived equations, indexation of GFR at extreme BMIs could have generated bias.

Overall, these findings align with the growing recognition that cystatin C is influenced by systemic inflammation, steroid exposure, and catabolic states [25–27]. Our data support the concept that cystatin C may also be a marker of frailty or illness and not a purely filtration-dependent biomarker. Accordingly, plasma cystatin C is used in the follow-up of several malignancies [28], and has been shown to be associated with metabolic syndrome [29], with neurological disorders and with frailty scores [30, 31]. Of note, in rare cases, increases in cystatin C that are not paralleled by an increase in creatinine may result not from cell metabolism and production of the tracer, but from differential renal clearance, despite the same GFR. This is illustrated by the shrunken pore syndrome [32] in which cystatin C equations underestimate creatinine derived GFR allegedly due to their differential filtration.

### Combined equations: benefits and limitations

Equations combining creatinine and cystatin C generally exhibited less bias, or at least a bias with a smaller magnitude. This can be explained either by the fact that in some situations, bias of creatinine and cystatin C-based equations were dependent on the same factor (inflammation) with opposite effects (like in patients with history of cirrhosis or kidney transplantation), and by the fact that the use of two biomarkers should lower the variability of the estimation. However, in several clinical scenarios (e.g. HIV infection, loop diuretic use, low or high BMI), the combined equation remained biased, particularly if the effect on the bias was in the same direction for creatinine-based and cystatin C-based equations like it was for younger age. These results reinforce the view that combined equations may mitigate—but not eliminate—bias, particularly when one biomarker is disproportionately affected. Of note, averaging two estimators may mathematically attenuate bias when the respective errors happen to be in opposite directions; however, this does not necessarily improve the intrinsic performance of the estimate. If one biomarker provides an accurate estimate while the other is substantially less reliable, combining the two may even reduce overall precision compared with using only the most accurate one. Therefore, the overall reduction in bias observed with combined equations should be interpreted cautiously, and knowledge of which tracer is expected to be more accurate depending on the individual clinical context is crucial.

### Impact on clinical practice

One main aspect of our findings is the cumulative aspect of the effects associated with clinical variables in the multivariable analysis. This means, that even the effect of one variable might seem low, or not clinically relevant, it must be considered with every other variable: for instance, a young woman with a low BMI and a kidney graft will have an overestimation of GFR between 23% and 30% with creatinine-based equations. By combining comorbidities (as illustrated in Table S3), the maximum theoretical negative bias could reach–35% with CKD-EPI_cys_ and the maximum theoretical positive bias was of +88% with MDRD_cr_ illustrating that in some specific situations GFR estimation may be very misleading for the clinician. Knowledge of these biases and their magnitude could guide clinician on which equation they should use (creatinine, cystatin C or combined) or even if a GFR measurement should be necessary.

### Strengths and limitations

This study benefits from a large and clinically diverse cohort, with a wide range of GFR, who underwent gold-standard, isotopic GFR and IDMS-traceable enzymatic creatinine and traceable cystatin C measurements.

However, our study has several limitations. It was conducted in a single center in France, potentially limiting generalizability. The use of 51Cr-EDTA and 99Tc-DTPA as GFR tracers and the use of urinary clearance differs from those used in CKD-EPI or MDRD development cohorts, potentially contributing to observed bias. Nonetheless, our study was designed to focus specifically on the differential bias across clinical variables rather than absolute bias values, mitigating concerns about the reference standard. The use of urinary clearance was justified by the evaluation of clinical variables known to be associated with a flawed plasma clearance such as cirrhosis, obesity, or low GFR.

Our sample, composed primarily of patients referred to a renal physiology unit, includes a high proportion of transplant recipients and patients with comorbidities, less represented in the training samples of existing equations, which is highly relevant to clinical practice.

## CONCLUSION

Our findings underscore the clinical relevance of bias in eGFR equations. We confirmed known sources of biomarker variability independent of renal glomerular filtration, identified new ones, and gave their respective weight in the estimation bias. These insights suggest that clinicians should interpret eGFR cautiously in certain contexts, and that combined equations, while generally more robust, are not immune to bias and cannot replace measured GFR.

Future equation development should consider incorporating clinical variables—such as medication use or comorbidities—alongside biomarkers to improve precision across patient populations.

## Supplementary Material

sfag157_Supplemental_File

## Data Availability

Data are not publicly available due to privacy and ethical restrictions in accordance with the European General Protection Data (GDPR). De-identified data may be shared by the corresponding author upon reasonable request for research purposes and in compliance with data protection regulations.
