# Supplementary material for: Clinical factors associated with biased estimation of glomerular filtration rate: a cross-sectional study
Source: Clin Kidney J. 2026 May 22;19(6):sfag157. doi: 10.1093/ckj/sfag157 (PMC13403280; doi:10.1093/ckj/sfag157)
Supplement: sfag157_Supplemental_File [file sfag157_Supplemental_File.docx]

# Supplemental Material

### Figure S1: Flowchart of the study

### TableS1: Formula of analyzed equations

### TableS2: Relative effect in % on eGFR/mGFR of each clinical parameter in uni and multivariable analyses, per equation including measured GFR.

### TableS3: Minimal and maximal impact on GFR estimation bias, according to different comorbidities.

**
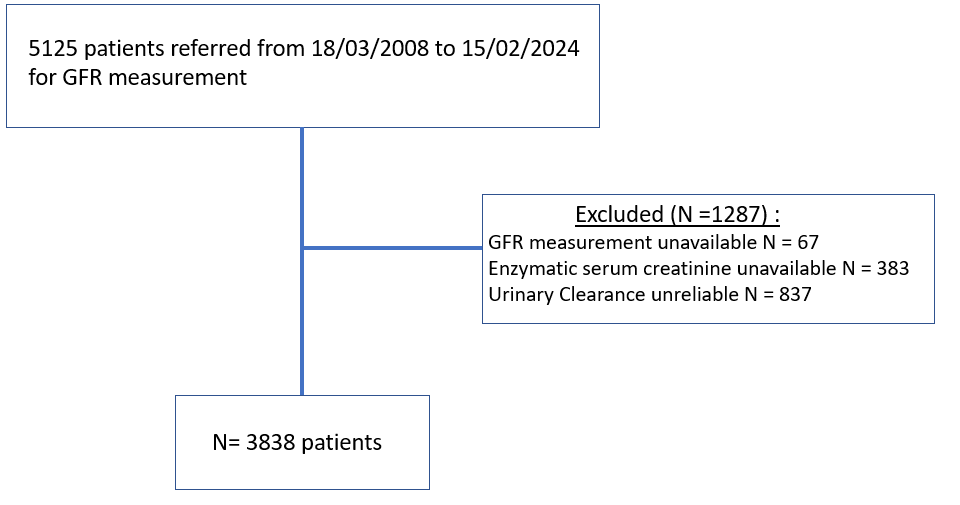
**

**Figure S1: Flowchart of the study**

| **Equation** | **Publication date** | **Formula** |
| --- | --- | --- |
| MDRD_cr_ | 1999 (simplified in 2006)  (2) | 186 ×(cr/88.4) ^-1.154^ ×age ^-0.203^ x0.742 (if female) ×1.212 (if black) |
| CKD-EPI_cr_ | 2009 (3) | 141xmin(cr/k,1)^α^×max(cr/k,1)^-1.209^×0.993^Age^×1.018^if female^×1.159^if black^  *k=80 for male, 62 for female; α = -0.411 for male, -0.329 for female* |
| CKD-EPI_cys_ | 2012 (4) | 133xmin(cys/0.8,1)^-0.499^×max(cys/0.8,1)^-1.328^×0.996^Age^×0.932 ^If female^) |
| CKD-EPI_cr_ | 2021 (5) | 142xmin(cr/k,1)^α^×max(cr/k,1)^-1.2^×0.9938^Age^×1.012^if female^  *k=80 for male, 62 for female; α = -0.302 for male, -0.241 for female* |
| CKD-EPI_cr-cys_ | 2021 (5) | 135xmin(cr/k,1)^α^×max(cr/k,1)^-0.544^×min(cys/0.8,1)^-0.323^×max(cys/0.8,1)^-0.778^×0.9961^Age^×0.963^if female^  *k=80 for male, 62 for female; α = -0.144 for male, -0.219 for female* |
| EKFC_cr_ | 2021 (6) | If age < 40: 107.3×min(cr/Q,1)^-0.322^×max(cr/Q,1)^-1.132^  If age > 40: 107.3×min(cr/Q,1)^-0.322^×max(cr/Q,1)^-1.132^×0.99^(Age-40)^  *Q= if age > 25: 80 for male (90 if sub-Saharan African origin), 62 for female* *(65 if sub-Saharan African origin)*  *If age < 25: for male: exp(3.2+0.259×age-0.543×log(age)-0.00763×age^2^+0.000079×age^3^)*  *For female: exp(3.2+0.259×age-0.543×log(age)-0.00763×age^2^+0.000079×age^3^)* |
| EKFC_cys_ | 2023 (7) | If age < 40: 107.3×min(cys/Q,1)^-0.322^×max(cys /Q,1)^-1.132^  If age > 40: 107.3xmin(cys /Q,1)^-0.322^×max(cys /Q,1)^-1.132^×0.99^(Age-40)^  *Q= if age < 50: 0.86 for male 0.79 for female ;*  *If age > 50: 0.86+0.005×(Age-50)*  for male ; *0.79+0.005×(Age-50)*  for female |
| EKFC_cr-cys_ | 2023 (7) | Mean of EKFC_cr_ and EKFC_cys_ |

**Table S1: Formulas of equations used to estimate GFR.**

MDRD: Modification of Diet in Renal Disease, CKD-EPI: Chronic Kidney Disease Epidemiology Collaboration, EKFC: European Kidney Function Consortium, cr : creatinine, cys : cystatin C

|  | **MDRD_cr_** | | **CKD EPI 2009_cr_** | | **CKD EPI 2021_cr_** | | **EKFC_cr_** | |
| --- | --- | --- | --- | --- | --- | --- | --- | --- |
|  | **Unadjusted effect, *% [99.9% CI]*** | **Adjusted effect, % [95% CI]** | **Unadjusted effect, *% [99.9% CI]*** | **Adjusted effect, % [95% CI]** | **Unadjusted effect, *% [99% CI]*** | **Adjusted effect, % [95% CI]** | **Unadjusted effect, *% [99.9% CI]*** | **Adjusted effect, % [95% CI]** |
| **Age 40-59 y** | Ref | Ref | Ref | Ref | Ref | Ref | Ref | Ref |
| **Age < 40 y** | 4.5 [ 2.6 ; 6.3 ] | 5.6 [ 3.8 ; 7.5 ] | 8.1 [ 6.3 ; 10.0 ] | 9.1 [ 7.2 ; 11.0 ] | 6.7 [ 4.8 ; 8.6 ] | 7.4 [ 5.6 ; 9.3 ] | 4.4 [ 2.6 ; 6.2 ] | 5.9 [ 4.1 ; 7.7 ] |
| **Age ≥ 60 y** | 5.7 [ 3.9 ; 7.6 ] | 3.1 [ 1.3 ; 5.0 ] | -0.2 [ -1.9 ; 1.5 ] | -2.5 [ -4.2 ; -0.7 ] | 2.7 [ 0.9 ; 4.5 ] | -1.2 [ -2.9 ; 0.6 ] | -3.9 [ -5.5 ; -2.3 ] | -7.5 [ -9.0 ; -6.0 ] |
| **Sex [male]** | Ref | Ref | Ref | Ref | Ref | Ref | Ref | Ref |
| **Sex [female]** | 4.0 [ 2.5 ; 5.6 ] | 3.9 [ 2.3 ; 5.4 ] | 5.9 [ 4.4 ; 7.5 ] | 5.6 [ 4.0 ; 7.1 ] | 5.5 [ 4.0 ; 7.1 ] | 5.0 [ 3.5 ; 6.5 ] | 2.2 [ 0.8 ; 3.7 ] | 1.9 [ 0.5 ; 3.3 ] |
| **Non-sub-Saharan African origin** | Ref | Ref | Ref | Ref | Ref | Ref | Ref | Ref |
| **Sub-Saharan African origin** | 3.4 [ 1.7 ; 5.2 ] | 6.7 [ 4.9 ; 8.5 ] | 0.0 [ -1.6 ; 1.8 ] | 2.5 [ 0.8 ; 4.2 ] | -13.6 [ -15.0 ; -12.1 ] | -11.4 [ -12.9 ; -9.9 ] | -3.3 [ -4.9 ; -1.7 ] | -1.6 [ -3.1 ; 0.0 ] |
| **BMI 18.5-29.9 kg/m²** | Ref | Ref | Ref | Ref | Ref | Ref | Ref | Ref |
| **BMI < 18.5kg/m²** | 15.1 [ 11.0 ; 19.3 ] | 13.3 [ 9.4 ; 17.4 ] | 15.8 [ 11.8 ; 20.0 ] | 12.0 [ 8.2 ; 15.9 ] | 15.2 [ 11.1 ; 19.5 ] | 11.6 [ 7.9 ; 15.5 ] | 14.2 [ 10.3 ; 18.1 ] | 10.5 [ 7.0 ; 14.2 ] |
| **BMI > 30 kg/m²** | -0.7 [ -2.5 ; 1.2 ] | -3.1 [ -4.9 ; -1.2 ] | -1.8 [ -3.6 ; -0.0 ] | -2.9 [ -4.7 ; -1.1 ] | -2.0 [ -3.8 ; -0.1 ] | -2.8 [ -4.6 ; -1.1 ] | -1.8 [ -3.5 ; -0.0 ] | -2.8 [ -4.5 ; -1.1 ] |
| **Absence of comorbidity/medication** | Ref | Ref | Ref | Ref | Ref | Ref | Ref | Ref |
| **Cirrhosis** | 19.2 [ 15.2 ; 23.3 ] | 17.8 [ 13.9 ; 21.8 ] | 15.3 [ 11.5 ; 19.3 ] | 16.9 [ 13.1 ; 20.8 ] | 18.8 [ 14.8 ; 22.9 ] | 16.5 [ 12.7 ; 20.3 ] | 16.5 [ 12.7 ; 20.3 ] | 15.7 [ 12.2 ; 19.4 ] |
| **HIV infection** | -8.1 [ -10.5 ; -5.8 ] | -5.5 [ -7.9 ; -3.1 ] | -8.3 [ -10.6 ; -6.0 ] | -4.6 [ -7.0 ; -2.2 ] | -11.2 [ -13.5 ; -8.9 ] | -4.4 [ -6.7 ; -2.0 ] | -8.6 [ -10.8 ; -6.4 ] | -4.3 [ -6.5 ; -2.0 ] |
| **Hypertension** | 2.3 [ 0.7 ; 3.9 ] | -1.6 [ -3.4 ; 0.2 ] | -0.4 [ -1.9 ; 1.1 ] | -1.7 [ -3.4 ; 0.1 ] | -0.3 [ -1.9 ; 1.3 ] | -1.4 [ -3.2 ; 0.3 ] | 1.5 [ 0.0 ; 3.1 ] | -1.6 [ -3.3 ; 0.1 ] |
| **Kidney transplant recipient** | 1.2 [ -0.3 ; 2.7 ] | 2.8 [ 0.4 ; 5.1 ] | 2.4 [ 0.8 ; 3.9 ] | 4.2 [ 1.9 ; 6.6 ] | 1.2 [ -0.3 ; 2.8 ] | 4.2 [ 1.9 ; 6.6 ] | 4.3 [ 2.8 ; 5.8 ] | 4.9 [ 2.7 ; 7.1 ] |
| **Diabetes** | 4.5 [ 2.6 ; 6.5 ] | 2.1 [ 0.1 ; 4.1 ] | 2.1 [ 0.3 ; 4.0 ] | 2.0 [ 0.0 ; 4.0 ] | 2.5 [ 0.6 ; 4.4 ] | 2.1 [ 0.1 ; 4.0 ] | 2.8 [ 1.0 ; 4.6 ] | 1.8 [ -0.0 ; 3.7 ] |
| **Corticosteroid use** | 1.7 [ 0.1 ; 3.3 ] | 1.4 [ -0.8 ; 3.7 ] | 2.3 [ 0.7 ; 3.9 ] | 1.5 [ -0.8 ; 3.7 ] | 0.8 [ -0.9 ; 2.4 ] | 1.4 [ -0.8 ; 3.7 ] | 3.5 [ 1.9 ; 5.1 ] | 1.5 [ -0.6 ; 3.6 ] |
| **Cotrimoxazole use** | -3.7 [ -5.6 ; -1.6 ] | -6.7 [ -8.9 ; -4.5 ] | -3.3 [ -5.2 ; -1.3 ] | -7.1 [ -9.2 ; -5.0 ] | -4.3 [ -6.3 ; -2.3 ] | -7.1 [ -9.2 ; -4.9 ] | -1.6 [ -3.6 ; 0.3 ] | -6.8 [ -8.8 ; -4.7 ] |
| **Loop Diuretic use** | 10.3 [ 7.8 ; 12.8 ] | 4.9 [ 2.4 ; 7.5 ] | 5.9 [ 3.5 ; 8.2 ] | 4.0 [ 1.6 ; 6.6 ] | 7.5 [ 5.1 ; 10.0 ] | 4.1 [ 1.7 ; 6.6 ] | 7.8 [ 5.5 ; 10.2 ] | 4.3 [ 1.9 ; 6.7 ] |
| **GFR > 60mL/min/1.73m²** | Ref | Ref | Ref | Ref | Ref | Ref | Ref | Ref |
| **GFR 30-60 mL/min/1.73m²** | 4.4 [ 2.9 ; 6.0 ] | 3.6 [ 1.7 ; 5.4 ] | 3.1 [ 1.5 ; 4.7 ] | 4.0 [ 2.2 ; 5.9 ] | 4.5 [ 2.9 ; 6.1 ] | 4.8 [ 3.0 ; 6.7 ] | 5.8 [ 4.3 ; 7.4 ] | 7.1 [ 5.3 ; 8.9 ] |
| **GFR < 30mL/min/1.73m²** | 20.3 [ 17.7 ; 23.0 ] | 16.6 [ 13.7 ; 19.6 ] | 14.5 [ 12.0 ; 17.1 ] | 13.9 [ 11.1 ; 16.7 ] | 17.8 [ 15.2 ; 20.4 ] | 15.4 [ 12.6 ; 18.3 ] | 21.3 [ 18.8 ; 23.8 ] | 21.8 [ 19.0 ; 24.7 ] |

**Panel A**

|  | **CKD EPI_cys_** | | **EKFC_cys_** | |
| --- | --- | --- | --- | --- |
|  | **Unadjusted effect, *% [99.9%]*** | **Adjusted effect, *% [95%]*** | **Unadjusted effect, *% [99.9%]*** | **Adjusted effect, *% [95%]*** |
| **Age 40-59 y** | Ref | Ref | Ref | Ref |
| **Age < 40 y** | 7.8 [ 5.6 ; 10.1 ] | 5.9 [ 3.7 ; 8.1 ] | 7.0 [ 4.9 ; 9.0 ] | 7.1 [ 5.2 ; 9.1 ] |
| **Age ≥ 60 y** | -6.3 [ -8.2 ; -4.4 ] | -6.5 [ -8.5 ; -4.5 ] | -2.5 [ -4.4 ; -0.7 ] | -6.7 [ -8.4 ; -4.9 ] |
| **Sex [male]** | Ref | Ref | Ref | Ref |
| **Sex [female]** | 6.0 [ 4.1 ; 7.9 ] | 4.3 [ 2.5 ; 6.1 ] | 2.4 [ 0.7 ; 4.0 ] | 1.3 [ -0.3 ; 2.8 ] |
| **Non-sub-Saharan African origin** | Ref | Ref | Ref | Ref |
| **Sub-Saharan African origin** | 3.0 [ 1.0 ; 5.1 ] | 7.3 [ 5.2 ; 9.5 ] | 3.0 [ 1.1 ; 4.9 ] | 6.2 [ 4.3 ; 8.1 ] |
| **BMI 18.5-29.9 kg/m²** | Ref | Ref | Ref | Ref |
| **BMI < 18.5kg/m²** | 6.7 [ 2.3 ; 11.3 ] | 3.0 [ -1.0 ; 7.3 ] | 8.9 [ 4.8 ; 13.1 ] | 3.5 [ -0.2 ; 7.2 ] |
| **BMI > 30 kg/m²** | -5.6 [ -7.7 ; -3.4 ] | -6.2 [ -8.2 ; -4.1 ] | -3.0 [ -5.0 ; -1.0 ] | -5.1 [ -7.0 ; -3.2 ] |
| **Absence of comorbidity/medication** | Ref | Ref | Ref | Ref |
| **Cirrhosis** | -8.5 [ -12.5 ; -4.4 ] | -9.0 [ -12.7 ; -5.1 ] | -2.9 [ -6.7 ; 1.1 ] | -7.1 [ -10.5 ; -3.6 ] |
| **HIV infection** | -2.1 [ -4.9 ; 0.7 ] | -6.3 [ -8.9 ; -3.7 ] | -3.6 [ -6.1 ; -1.0 ] | -4.8 [ -7.1 ; -2.5 ] |
| **Hypertension** | -10.7 [ -12.2 ; -9.1 ] | -2.8 [ -4.9 ; -0.8 ] | -3.1 [ -4.6 ; -1.5 ] | -1.4 [ -3.2 ; 0.5 ] |
| **Kidney transplant recipient** | -12.1 [ -13.7 ; -10.6 ] | -7.8 [ -10.7 ; -4.9 ] | -5.4 [ -7.0 ; -3.9 ] | -5.5 [ -8.1 ; -2.7 ] |
| **Diabetes** | -8.9 [ -10.8 ; -6.9 ] | -3.0 [ -5.2 ; -0.7 ] | -3.2 [ -5.1 ; -1.3 ] | -2.7 [ -4.7 ; -0.7 ] |
| **Corticosteroid use** | -12.9 [ -14.5 ; -11.3 ] | -8.7 [ -11.4 ; -5.9 ] | -6.8 [ -8.4 ; -5.2 ] | -7.3 [ -9.7 ; -4.8 ] |
| **Cotrimoxazole use** | -9.7 [ -11.9 ; -7.5 ] | 0.5 [ -2.4 ; 3.3 ] | -4.4 [ -6.6 ; -2.3 ] | 0.3 [ -2.2 ; 2.9 ] |
| **Loop diuretic use** | -5.7 [ -8.5 ; -2.9 ] | -1.6 [ -4.6 ; 1.6 ] | 4.8 [ 2.0 ; 7.7 ] | 0.5 [ -2.3 ; 3.4 ] |
| **GFR > 60mL/min/1.73m²** | Ref | Ref | Ref | Ref |
| **GFR 30-60 mL/min/1.73m²** | -7.3 [ -9.0 ; -5.6 ] | 2.6 [ 0.4 ; 4.8 ] | 2.2 [ 0.6 ; 3.9 ] | 11.1 [ 9.0 ; 13.2 ] |
| **GFR < 30mL/min/1.73m²** | 2.0 [ -0.9 ; 4.9 ] | 13.9 [ 10.3 ; 17.6 ] | 23.8 [ 20.8 ; 27.0 ] | 35.7 [ 32.0 ; 39.6 ] |

**Panel B**

|  | **CKD EPI_cr-cys_** | | **EKFC_cr-cys_** | |
| --- | --- | --- | --- | --- |
|  | **Unadjusted effect, *% [99.9%]*** | **Adjusted effect, *% [95%]*** | **Unadjusted effect, *% [99.9%]*** | **Adjusted effect, *% [95%]*** |
| **Age 40-59 y** | Ref | Ref | Ref | Ref |
| **Age < 40 y** | 5.6 [ 3.8 ; 7.4 ] | 4.4 [ 2.6 ; 6.2 ] | 5.6 [ 3.8 ; 7.4 ] | 6.4 [ 4.8 ; 8.2 ] |
| **Age ≥ 60 y** | -1.0 [ -2.7 ; 0.7 ] | -2.1 [ -3.8 ; -0.4 ] | -2.2 [ -3.8 ; -0.6 ] | -6.7 [ -8.2 ; -5.2 ] |
| **Sex [male]** | Ref | Ref | Ref | Ref |
| **Sex [female]** | 6.2 [ 4.7 ; 7.7 ] | 5.0 [ 3.5 ; 6.5 ] | 2.2 [ 0.8 ; 3.6 ] | 1.6 [ 0.3 ; 2.9 ] |
| **Non-sub-Saharan African origin** | Ref | Ref | Ref | Ref |
| **Sub-Saharan African origin** | -5.4 [ -6.9 ; -3.8 ] | -1.7 [ -3.3 ; -0.1 ] | -0.6 [ -2.2 ; 1.0 ] | 2.1 [ 0.6 ; 3.7 ] |
| **BMI 18.5-29.9 kg/m²** | Ref | Ref | Ref | Ref |
| **BMI < 18.5kg/m²** | 9.1 [ 5.4 ; 12.9 ] | 6.3 [ 2.8 ; 9.9 ] | 12.6 [ 9.0 ; 16.4 ] | 7.3 [ 4.0 ; 10.6 ] |
| **BMI > 30 kg/m²** | -4.3 [ -6.0 ; -2.5 ] | -5.3 [ -7.0 ; -3.5 ] | -2.3 [ -4.1 ; -0.6 ] | -4.4 [ -6.0 ; -2.7 ] |
| **Absence of comorbidity/medication** | Ref | Ref | Ref | Ref |
| **Cirrhosis** | 3.4 [ -0.3 ; 7.2 ] | 1.1 [ -2.3 ; 4.7 ] | 7.8 [ 4.1 ; 11.6 ] | 3.9 [ 0.7 ; 7.3 ] |
| **HIV infection** | -5.9 [ -8.0 ; -3.7 ] | -5.9 [ -8.0 ; -3.7 ] | -6.0 [ -8.1 ; -3.9 ] | -4.6 [ -6.6 ; -2.6 ] |
| **Hypertension** | -7.4 [ -8.7 ; -6.1 ] | -2.5 [ -4.2 ; -0.8 ] | -0.8 [ -2.2 ; 0.6 ] | -1.5 [ -3.0 ; 0.1 ] |
| **Kidney transplant recipient** | -8.8 [ -10.1 ; -7.4 ] | -4.8 [ -7.3 ; -2.3 ] | -1.2 [ -2.6 ; 0.2 ] | -2.1 [ -4.4 ; 0.3 ] |
| **Diabetes** | -4.5 [ -6.1 ; -2.8 ] | -0.8 [ -2.7 ; 1.1 ] | 0.3 [ -1.4 ; 2.0 ] | -0.6 [ -2.4 ; 1.2 ] |
| **Corticosteroid use** | -8.9 [ -10.2 ; -7.5 ] | -4.2 [ -6.6 ; -1.8 ] | -1.8 [ -3.3 ; -0.3 ] | -2.4 [ -4.6 ; -0.1 ] |
| **Cotrimoxazole use** | -8.7 [ -10.5 ; -6.9 ] | -2.6 [ -4.9 ; -0.3 ] | -2.8 [ -4.7 ; -0.9 ] | -2.9 [ -5.0 ; -0.8 ] |
| **Loop Diuretic use** | 0.4 [ -2.0 ; 2.8 ] | 2.2 [ -0.5 ; 4.9 ] | 8.9 [ 6.4 ; 11.4 ] | 4.4 [ 1.9 ; 6.9 ] |
| **GFR > 60mL/min/1.73m²** | Ref | Ref | Ref | Ref |
| **GFR 30-60 mL/min/1.73m²** | -3.5 [ -5.0 ; -2.1 ] | 2.2 [ 0.5 ; 4.1 ] | 4.5 [ 3.1 ; 5.9 ] | 10.1 [ 8.3 ; 11.9 ] |
| **GFR < 30mL/min/1.73m²** | 5.8 [ 3.3 ; 8.2 ] | 10.2 [ 7.4 ; 13.2 ] | 24.9 [ 22.3 ; 27.6 ] | 30.5 [ 27.4 ; 33.7 ] |

**Panel C**

**Table S2: Relative effect in % on eGFR/mGFR of each clinical parameter in uni and multivariable analyses, per equation including measured GFR.**

Panel A: Creatinine-based equations, Panel B: Cystatin C-based equations, Panel C: Creatinine and cystatin C-based equations.

The unadjusted effect represents the exponential of the regression coefficient minus 1 associated with the variable of the linear regression of log[eGFR/mGFR] in an univariable analysis, and the adjusted effect represents the exponential of the regression coefficient minus 1 with the variable of the linear regression of log [eGFR/mGFR] in a multivariable analysis. In the multivariable model, covariates were to age, sex, ethnicity, BMI, cirrhosis, hypertension, treated diabetes, HIV, kidney transplant recipients, use of corticosteroids, cotrimoxazole, loop diuretics and measured GFR by categories.

*grey cases highlight that confidence interval does not include 0

| **Equation** | **Bias** | | **Age** | **Sex** | **Ethnic** | **BMI** | **Cirrhosis** | **HIV** | **HTN** | **KT** | **Diabetes** | **Corticosteroids** | **Cotrimoxazole** | **Loop Diuretrics** |
| --- | --- | --- | --- | --- | --- | --- | --- | --- | --- | --- | --- | --- | --- | --- |
| **MDRD_cr_** | Min | **-15 %** | 40-60 | M | No S-SAO | >30 | N | Y | N | N | N | No impact | Y | N |
|  | Max | **+87.8%** | <40 | F | S-SAO | <18.5 | Y | N | Y | Y | Y |  | N | Y |
| **CKD_cr_** 09 | Min | **-14.8%** | >40 | M | No S-SAO | >30 | N | Y | No impact | N | N |  | Y | N |
|  | Max | **+84.7%** | <40 | F | S-SAO | <18.5 | Y | N |  | Y | Y |  | N | Y |
| **CKD_cr_** 21 | Min | **-24.6%** | >40 | M | S-SAO | >30 | N | Y |  | N | N |  | Y | N |
|  | Max | **+77.9%** | <40 | F | No S-SAO | <18.5 | Y | N |  | Y | Y |  | N | Y |
| **EKFC_cr_** | Min | **-19.4%** | >60 | M | S-SAO | >30 | N | Y |  | N | N |  | Y | N |
|  | Max | **+60%** | <40 | F | No S-SAO | <18.5 | Y | N |  | Y | Y |  | N | Y |
| **CKD_cys_** | Min | **-35.2%** | >60 | M | No S-SAO | >30 | Y | Y | N | Y | No impact | Y | N | N |
|  | Max | **+24%** | <40 | F | S-SAO | <18.5 | N | N | N | N |  | N | N | N |
| **EKFC_cys_** | Min | **-22%** | >60 | M | No S-SAO | >30 | N | Y | N | Y |  | Y | N | N |
|  | Max | **+39.6%** | <40 | F | S-SAO | <18.5 | N | N | Y | N |  | N | N | Y |
| **CKD_cr-cys_** | Min | **-22%** | >40 | M | S-SAO | >30 | N | Y | N | Y |  | Y | Y | N |
|  | Max | **+24.5%** | <40 | F | No S-SAO | <18.5 | N | N | N | N |  | N | N | Y |
| **EKFC_cr-cys_** | Min | **-15.9%** | >60 | M | No impact | >30 | N | Y | N | No impact |  | N | Y | N |
|  | Max | **+35.6%** | <40 | F |  | <18.5 | Y | N | Y |  |  | N | N | Y |

**Table S3:** **Minimal and maximal impact on GFR estimation bias, according to different combinations of comorbidities, for all equations.**

MDRD: Modification of Diet in Renal Disease, CKD-EPI: Chronic Kidney Disease Epidemiology Collaboration, EKFC: European Kidney Function Consortium, cr: creatinine, cys: cystatin C, BMI: Body Mass Index; HIV: Human Immunodeficiency Virus, HTN: Hypertension; M: Male, F: Female, S-SAO: Sub-saharan African Origin, Y: Yes, N: No.
